# Prognostic Performance of Ten Liver Function Models in Patients with Hepatocellular Carcinoma Undergoing Radiofrequency Ablation

**DOI:** 10.1038/s41598-018-19251-y

**Published:** 2018-01-16

**Authors:** Shu-Yein Ho, Po-Hong Liu, Chia-Yang Hsu, Yi-You Chiou, Chien-Wei Su, Yun-Hsuan Lee, Yi-Hsiang Huang, Fa-Yauh Lee, Ming-Chih Hou, Teh-Ia Huo

**Affiliations:** 1 Department of Medicine, Taipei Veterans General Hospital, Taipei, Taiwan; 2 Department of Radiology, Taipei Veterans General Hospital, Taipei, Taiwan; 3 Faculty of Medicine, National Yang-Ming University School of Medicine, Taipei, Taiwan; 4 Institute of Clinical Medicine, National Yang-Ming University School of Medicine, Taipei, Taiwan; 5 Institute of Pharmacology, National Yang-Ming University School of Medicine, Taipei, Taiwan; 6 Harvard T.H. Chan School of Public Health, Boston, MA USA; 7 Department of Internal Medicine, University of Nevada School of Medicine, Reno, NV USA

## Abstract

Liver functional capacity is a crucial survival determinant for hepatocellular carcinoma (HCC). Noninvasive models were proposed to assess hepatic reserve, but their performance in outcome prediction is unclear. We aimed to investigate 10 currently used liver function models in HCC patients undergoing radiofrequency ablation (RFA). A total 499 HCC patients were prospectively identified. Homogeneity and corrected Akaike information criteria (AICc) were compared. Cox proportional hazards model was used to identify independent survival predictors. Significance survival differences were found across 10 noninvasive models (all p < 0.001) except for GUCI and APRI grade 2 vs 3, and King’s score grade 1 vs 2. Among these models, ALBI grade showed the highest homogeneity and lowest AICs value, indicating a better prognostic performance. Within Child-Turcotte-Pugh (CTP) score 5 group, significant survival difference was demonstrated between ALBI grade 1 and 2 (p < 0.001); for those with CTP score 6 or higher, only ALBI grade 2 and 3 showed survival difference (p < 0.001). Cox analysis disclosed that ALBI grade, tumor size and performance status were independent prognostic predictors. There was significant correlation between CTP score and other 9 models. We conclude that ALBI grade may serve as objective and feasible surrogate for prognostic prediction in HCC patients undergoing RFA.

## Introduction

Hepatocellular carcinoma (HCC) is one of the most common malignancies worldwide, accounting for nearly 782,500 new cases and 745,500 deaths worldwide during 2012^1^. Despite improved diagnostic and therapeutic efforts, the mortality from HCC is increasing^2^. Hepatitis B virus (HBV) infection, hepatitis C virus (HCV) infection and alcohol consumption are well-established major risk factors for HCC^3,4^. According to HCC management guidelines endorsed by the American Association for the Study of Liver Diseases (AASLD) and European Association for the Study of the Liver (EASL), the major treatment options for HCC are surgical resection, liver transplantation, local ablative therapies such as percutaneous ethanol injection and radiofrequency ablation (RFA), transarterial chemoembolization and targeted therapy^5,6^. For early stage HCC, surgical resection and liver transplantation are considered cornerstone of curative treatments. Among local ablative therapies, RFA is usually the treatment of choice for small HCC and may provide 5-year survival rates up to 50–70%^7^.

The prognosis and management of HCC depend on tumor burden, performance status and patient’s liver function^8^, the last usually being characterized by the Child-Turcotte-Pugh (CTP) classification. CTP classification has been incorporated into most HCC staging systems including Barcelona Clinic Liver Cancer (BCLC) system, the Cancer of Liver Italian Program (CLIP) score and Japan Integrated staging (JIS) to assess the severity of liver dysfunction^9^. However, CTP classification has several drawbacks including arbitrary use of cutoff values, the same weighted risk among different parameters, and subjective assessment of ascites and hepatic encephalopathy^10,11^.

HCC patients may present from chronic hepatitis with normal liver function to early or advanced liver cirrhosis accompanied by hepatic decompensation^12^. Liver biopsy is the gold standard to assess liver fibrosis but is associated with complications such as pain, bleeding, bile duct injury, or penetration of abdominal viscera. Also, sampling errors and inter-observer variation may decrease its reliability^13^. Recently, various noninvasive liver function models were proposed to assess the severity of hepatic injury^14^. The albumin-bilirubin (ALBI) grade, based solely on serum albumin and bilirubin level^15–17^, and platelet-albumin-bilirubin (PALBI) grade, which include serum albumin, bilirubin and platelet count, are useful markers of liver reserve in HCC^16^. The model for end-stage liver disease (MELD) which is based on serum bilirubin, creatinine and prothrombin, is also used to assess liver dysfunction in HCC^18–20^. Alternative tools to evaluate liver functions are fibrosis index based on 4 factors (FIB-4) and aspartate aminotransferase-to- platelet ratio (APRI)^21–26^; these models comprise clinical parameters such as age and serum biochemistry. Other models, including Lok index^14,27^, cirrhosis discriminant index (CDS)^28^, King’s score^29,30^, and Göteborg University Cirrhosis Index (GUCI)^31^, are also used; these models can be readily calculated from clinical variables such as age, serum aspartate aminotransferase, alanine aminotransferase, international normalized ratio of prothrombin (INR) and platelet count. To date, at least ten noninvasive liver function models have been used to define the severity of hepatic dysfunction. However, the performance of these models to predict the prognosis of HCC is unclear. This study aimed to compare these ten models in outcome prediction for HCC patients undergoing RFA.

## Methods

### Patients

Patients with a confirmed diagnosed of HCC and admitted to Taipei Veterans General Hospital during the period from 2002 to 2013 were prospectively identified and retrospectively analyzed. A total 499 patients who received RFA as the primary treatment were enrolled. Comprehensively baseline information, including patient’s demographics, etiology of liver disease, performance status, tumoral status, serum biochemistry, noninvasive liver reserve models and grading, were recorded at the times of diagnosis. Patients were followed every 3–6 months until death or dropout from follow-up. This study was approved by the Institutional Review Broad at Taipei Veterans General Hospital and complies with the standards of the Declaration of Helsinki and current ethical guidelines. Waiver of consent was obtained, and patient records/information was anonymized and de-identified prior to analysis.

### Diagnosis and definition

The diagnosis of HCC was histologically confirmed by needle biopsy or based on the findings of typical radiological features in at least two imaging examinations including sonography, contrast-enhanced dynamic computed tomography (CT), magnetic resonance imaging (MRI), and hepatic arterial angiography^32^.The performance status was assessed by using the Eastern Cooperative Oncology Group Performance scaling ranging from 0 (asymptomatic) to 4 (confined to bed). HBV-related HCC was defined as seropositive for hepatitis B surface antigen (HBsAg), seronegative for anti-HCV antibody and no history of alcoholism. Patients who were seropositive for anti-HCV antibody, seronegative for HBsAg and without history of alcoholism were classified as HCV-related HCC. Dual HBV- and HCV-related HCC was defined as seropositive for HBsAg and anti-HCV antibody. Total tumor volume (TTV) was calculated as the sum of all tumor nodule volumes, and each tumor volume was calculated as 4/3 × 3.14 × (maximum radius of the tumor in cm)^3^, as previously described^33^.The ten liver function models were calculated according to their original formula, and grading of severity was classified according to the score distribution^15,16,19,21,26,28,29,31^. Regarding about the severity of grading system, grade 1 represents the best liver function and grade 3 indicates the worst liver function.

### Treatment

The newly diagnosed HCC patients at Taipei Veterans General Hospital were discussed in multidisciplinary meetings that included hepatologists, oncologists, surgeons, pathologists and radiologists for the diagnosis and treatment strategy. The criteria for RFA included (1) early stage HCC (single tumor ≤5 cm in diameter, or tumor number ≤3, maximum diameter of each ≤3 cm), (2) no extrahepatic metastasis or major vascular invasion, (4) platelet count greater than 50,000/mm^3^, and (5) refusal of surgical treatment. Therapeutic information including benefits and risks was provided to individual patient based on shared decision-making. Written informed consent was obtained prior to initiation of treatment.

RFA was performed according to the standard procedure^34^. Under ultrasound guidance, the tumor(s) was ablated by using a 17-gauge, cooled-tip electrode with the Cool-Tip Radiofrequency System (Radionics, Burlington, MA, USA). The ablation was performed in automatic impedance control mode in which the current output was automatically adjusted. Post-RFA sonography was performed immediately to confirm that there was no definite hemorrhage or hematoma.

### Statistics

The Chi-square test and two-tailed Fisher’s exact test were used for categorical data, and Mann–Whitney U test was used to compare continuous variables. Missing values were handled by multiple imputation while a complete case was used as benchmark analysis^35^. Data are expressed as the mean ± standard deviation (SD) and median with interquartile range. The overall survival was estimated by the Kaplan-Meier method and compared by log-rank test. Factors possibly associated with survival, including age, sex, etiology of liver disease, severity of liver cirrhosis, size and number of tumor nodules, serum biochemistry, performance status, and noninvasive liver reserve models were analyzed. Factors that were significant in the univariate survival analysis were introduced into the multivariate Cox proportional hazards model to determine the adjusted hazard ratios (HR) and 95% confidence intervals (CI)^36^.

The discriminatory ability of different liver function models to predict survival was examined by using the Cox proportional hazards model, and the consequences of the Cox model were expressed with the corrected Akaike information criterion (AICc), which reveals how the model affects the dependent variable (patient survival) and represents an overall assessment of the model^37,38^. The lower the AIC, the more explanatory and informative the model is^39^. We also analyzed correlation of CTP scores and other 9 noninvasive liver function models. Statistical significance levels were determined by two-tailed tests. A *p* value < 0.05 was considered statistically significant. All statistical analyses were conducted using the SPSS for Windows version 21 release (SPSS Inc., Chicago, IL, USA).

## Results

Between 2002 and 2013, a total 499 patients who received RFA were enrolled. As shown in Table 1, the median age was 67 years and the majority of patients were male (65%). The etiology of HCC was hepatitis B in 183 (37%), hepatitis C 160 (32%), dual hepatitis B and C in 19 (4%), alcoholism in 81 (16%) and others in 75 (15%). A total of 143 (29%) patients had diabetes mellitus, 446 (89%) patients had performance status 0 to 1, and 69 (14%) patients had presence of ascites at the time of diagnosis. Three hundred and eighty-four (77%) patients had a single tumor at presentation and 206 (41%) patients had maximum tumor diameter <2 cm. The calculation formula and severity of grading of the 10 models are described in Table 2.

**Table 1:** Baseline characteristics of patients of hepatocellular carcinoma undergoing radiofrequency ablation (RFA).

Variables	Patients (n = 499)
Age (years, median [interquartile range])	67 [58–76]
Male, n (%)	326 (65)
Etiologies of liver disease	
HBV, n (%)	183 (37)
HCV, n (%)	160 (32)
HBV + HCV, n (%)	19 (4)
Alcohol, n (%)	81 (16)
Others, n (%)	75 (15)
^a^Diabetes mellitus, n (%)	143 (29)
Performance status (0/1/2-3), n (%)	370/76/53 (74/15/11)
Ascites, n (%)	69 (14)
Alpha-fetoprotein(ng/ml, median, [interquartile range])	18.24 [6.93–67.67]
Laboratory values (mean ± SD)	
Alanine aminotransferase (IU/L)	64.47 ± 55.04
Aspartate aminotransferase (IU/L)	64.46 ± 52.88
^a^Alkaline phosphatase (IU/L)	107.97 ± 78.85
Albumin (g/dl)	3.75 ± 0.6
Total bilirubin (mg/dl)	1.08 ± 1.06
Creatinine (mg/dl)	1.184 ± 1.154
Platelets (1,000/μL)	125.54 ± 62.13
INR of prothrombin time	1.09 ± 0.14
Non-invasive liver function models	
ALBI grade (1/2/3), n (%)	209/261/29 (42/52/6)
APRI grade (1/2/3), n (%)	122/205/172(24/41/35)
CTP classification (A/B/C), n (%)	413/76/10 (83/15/2)
CDS grade (1/2/3), n (%)	78/320/101 (16/64/20)
FIB-4 grade (1/2/3), n (%)	37/138/324 (7/28/65)
GUCI grade (1/2/3), n (%)	119/199/181 (24/30/46)
Lok index grade (1/2/3), n (%)	204/196/99 (41/39/20)
MELD score (1/2/3), n (%)	217/197/85 (43/40/17)
PALBI grade (1/2/3), n (%)	273/157/69 (55/31/14)
King’s score (1/2/3), n (%)	31/112/356 (6/22/72)
Tumor nodules (1/2/ ≥ 3), n (%)	384/79/36 (77/16/7)
Maximal tumor diameter (≤2/2-3/>3 cm), n (%)	206/172/121 (41/35/24)
TTV (ml, median, [interquartile range])	7.24 [2.57–16.25]

ALBI, Albumin-bilirubin; APRI, Aspartate transaminase-to-Platelet ratio;

CDS, Cirrhosis discriminant index; CTP, Child-Turcotte-Pugh score; FIB-4, Fibrosis-4 score; HBV, hepatitis B virus; HCV, hepatitis C virus; MELD, Model for End-stage liver disease; GUCI, Göteborg University Cirrhosis Index; PALBI, platelet-albumin-bilirubin; TTV, total tumor volume; SD, standard deviation;

^a^Missing of data of DM and alkaline phosphatase were 3 (0.6%) and 28 (5.6%) patients, respectively.

**Table 2:** Formula and grading of noninvasive liver function models.

Noninvasive liver function models	Formula
ALBI, Grade 1/2/3 (<−2.6/−2.6- ≤ −1.39 />−1.39)	(log(Bilirubin[μmol/L]) × 0.66) − (Albumin[g/L] × 0.085)
APRI, Grade 1/2/3 (<0.5/0.5–1.5/> 1.5)	AST (/UNL)/platelet (10^9^/L) × 100
CTP, A/B/C (5-6/7–9/10–15)	Encephalopathy: none = 1, grade 1 or 2 = 2, grade 3 or 4 = 3 Ascites: none = 1, mild to moderate = 2, severe = 3 Bilirubin(mg/dl): <2 = 1, 2-3 = 2, >3 = 3 Albumin(g/dl): >3.5 = 1, 2.8–3.5 = 2, <2.8 = 3 PTsec (INR): <4(1.7) = 1, 4–6(1.7–2.3) = 2, >6(>2.3) = 3
CDS, Grade 1/2/3(<4/4–7/>7)	Platelet count (×10^9^/L): >340 = 0; 280–339 = 1; 220–279 = 2; 160–219 = 3; 100–159 = 4; 40–99 = 5; <40 = 6 ALT/AST ratio: >1.7 = 0; 1.2–1.7 = 1; 0.6–1.19 = 2; <0.6 = 3 INR: <1.1 = 0; 1.1–1.4 = 1; >1.4 = 2 CDS is the sum of the above (possible value 0–11)
FIB-4 index, Grade 1/2/3 (<1.45/1.45–3.25/>3.25)	Age × AST/[Platelet × (ALT)^1/2^]
GUCI, Grade 1/2/3 (<0.5/0.5–1.56/>1.56)	AST/TOPNORMAL AST × INR ×100/(Platelets × 10^9^)
Lok index, Grade 1/2/3 (<0.5/0.5–0.8/>0.8)	Lok Index = e^(LogOddsLok)^/(1 + e^(LogOddsLok)^) Log Odds Lok = (1.26 × AST/ALT) + (5.27 × INR) - (0.0089 × Platelets × 10^9^) − 5.56
MELD, Grade 1/2/3 (<8/8–12/>12)	10 × ((0.957 × ln(Creatinine)) + (0.378 × ln(Bilirubin)) + (1.12 × ln(INR))) + 6.43
PABLI, Grade1/2/3 (≤−2.53, −2.53 and ≤ −2.09, >−2.09)	2.02 × log_10_ bilirubin [umol/L] − 0.37 × (log_10_ bilirubin)^2^ − 0.04 × albumin[g/L] − 3.48 × log_10_ platelets[× 10^9^/L] + 1.01 × (log_10_ platelets[× 10^9^/L])
King’s score Grade 1/2/3 (<7.6/7.6–16.7/>16.7)	Age × AST × INR/[platelets (x10^9^/L)]

ALBI, Albumin-bilirubin; APRI, Aspartate transaminase-to-Platelet ratio;

CDS, Cirrhosis discriminant index; CTP, Child-Turcotte-Pugh score; FIB-4, Fibrosis-4 score; FCI, Fibrosis-cirrhosis index; FI, fibrosis index; HBV, hepatitis B virus; HCV, hepatitis C virus; MELD, Model for End-stage liver disease; GUCI, Göteborg University Cirrhosis Index;

PALBI, platelet-albumin-bilirubin; SD, standard deviation.

Among possible predictors of survival, there were 28 (5.6%) patients who did not have baseline value of serum alkaline phosphatase (alk-P). Logistic regression on missing data indicators using completely observed variables as covariates was implemented. The statistical output obtained from multiple imputation was similar to statistical output from a complete case analysis in which patients with missing data were omitted. Pooled results by multiple imputation were reported in this study.

**Figure 1:**
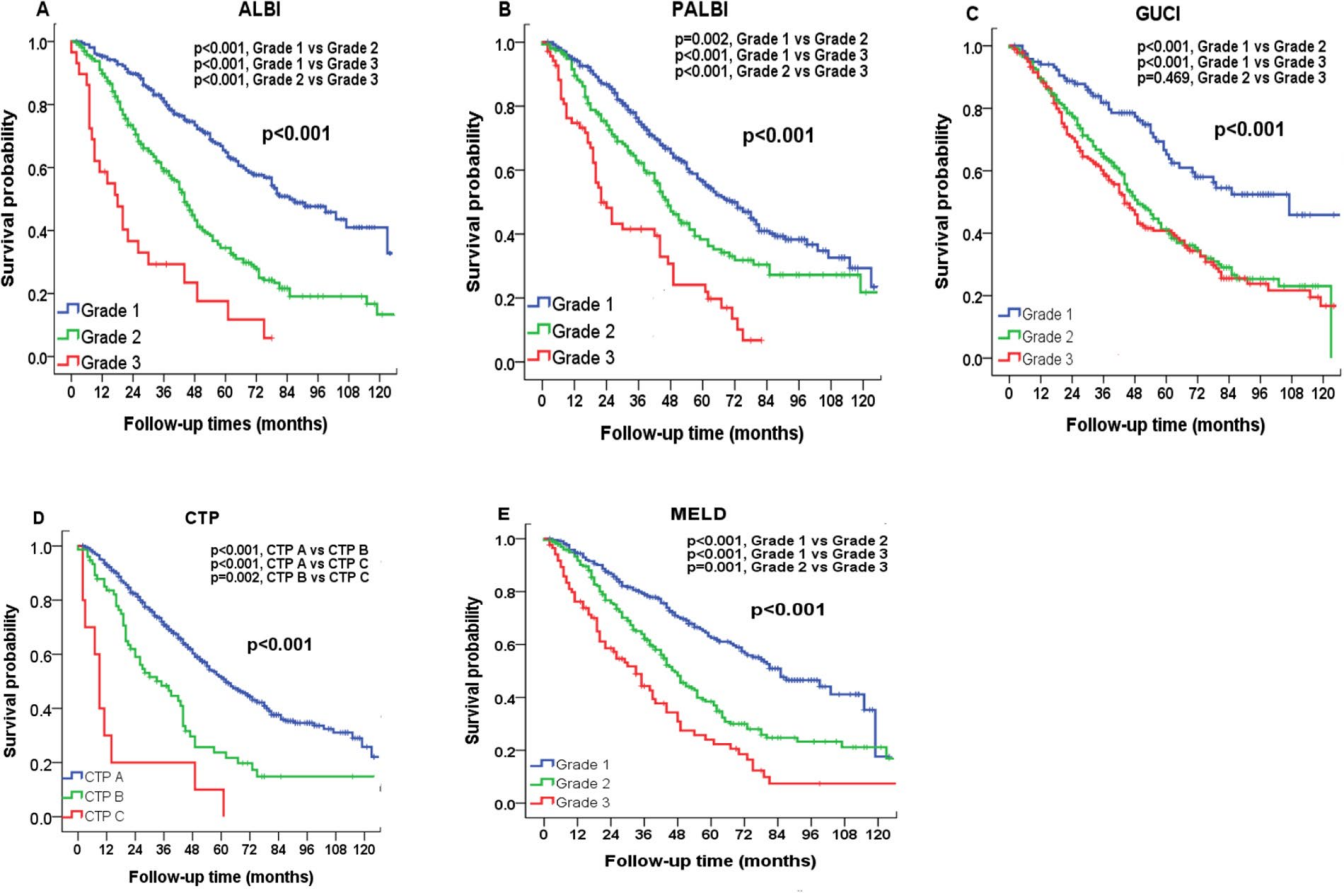
Comparison of survival distribution according to (**A**) ALBI, (**B**) PALBI, (**C**) GUCI, (**D**) CTP, and (**E**) MELD grading. Significant survival differences are found in all 5 models.

**Figure 2:**
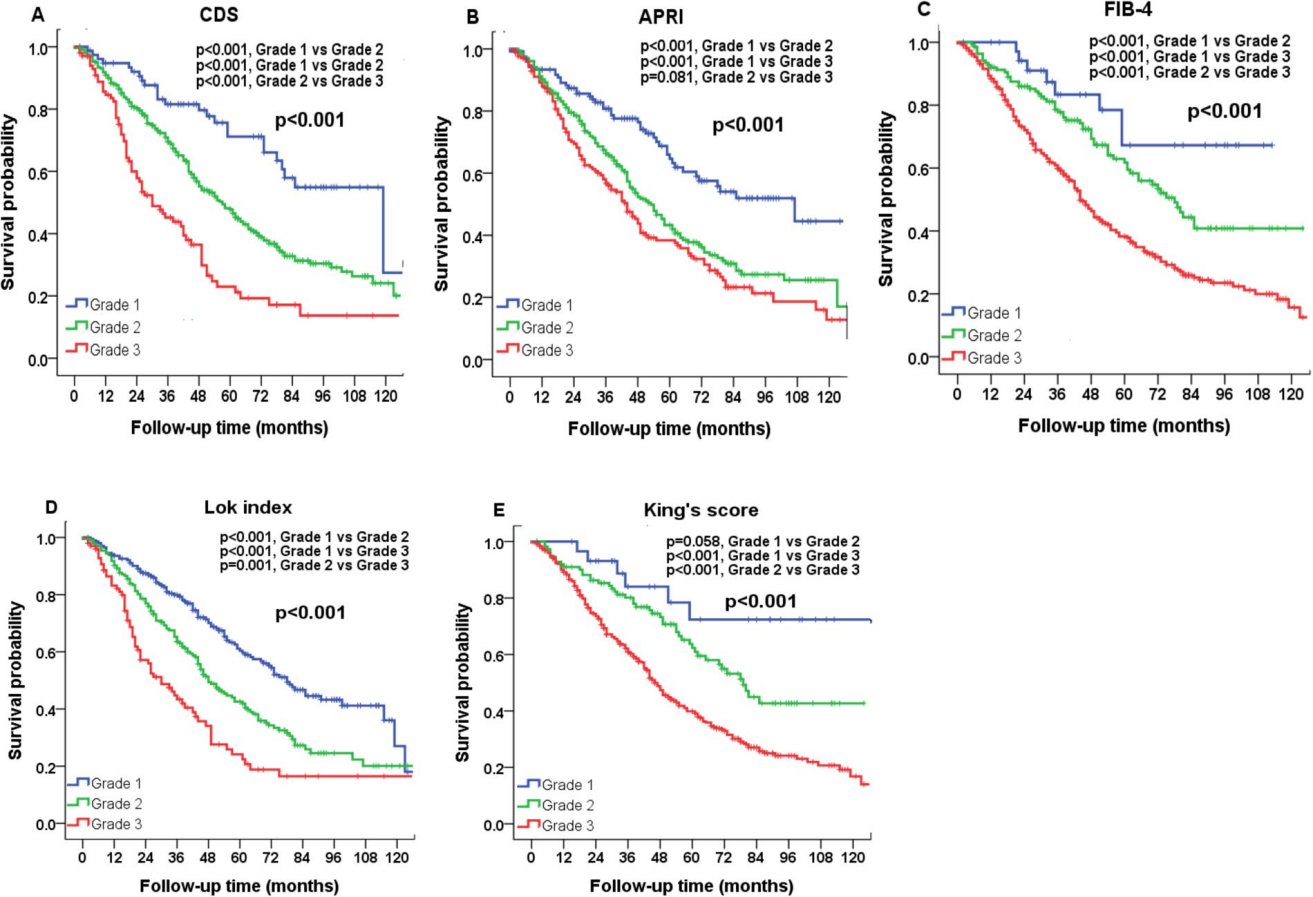
Comparison of survival distributions according to (**A**) CDS, (**B**) APRI, (**C**) FIB-4 index, (**D**) Lok index, and (**E**) King’s score grading. Significant survival differences are found in all 5 models.

The median and mean follow-up period was 47 months and 42 months, respectively. The overall survival rate at 1-year, 3-year and 5-year were 90.4%, 68.5% and 53.5%. The survival distributions according to ALBI, PALBI, GUCI, CTP, MELD, CDS, APRI, FIB-4, Lok-index and King’s score are shown in Figs 1 and 2. Significant survival differences were found across all strata in each liver function model except for GUCI grade 2 vs 3 (*p* = 0.469), APRI grade 2 vs 3 (*p* = 0.081) and King’s score grade 1 vs 2 (*p* = 0.058).

The prognostic performance of the ten liver function models was compared. Of these models, ALBI grade has the highest homogeneity and lowest AIC value, followed by CDS and MELD (Table 3). The prognostic role of the ALBI score was analyzed along with other clinically important prognostic predictors. In univariate survival analysis, presence of ascites, aspartate aminotransferase, alkaline phosphatase, international normalized ratio (INR) of prothrombin time (PT), serum platelet count, serum AFP level, tumor size, total tumor volume, performance status and ALBI grade were the factors associated with prognosis (Table 4; all *p* values < 0.05). Cox multivariate analysis revealed that tumor size >2 cm (hazard ratio [HR]: 1.635, 95% confidence interval [CI]: 1.269–2.106, *p* < 0.001), poor performance status (HR: 1.788, 95% CI: 1.349–2.369, *p* < 0.001), ALBI grade 2 (HR: 2.352, 95% CI: 1.786–3.098, *p* < 0.001) and grade 3 (HR: 4.720, 95% CI: 2.905–7.669, *p* < 0.001) were independent predictors associated with a decreased survival. The correlation between CTP score and other 9 liver function models was further investigated. The scores of the 9 models increased with increasing CTP scores, suggesting a worsened liver functional reserve (Table 5).

**Table 3:** Comparison of prognostic performance of noninvasive liver function models for radiofrequency ablation.

	Homogeneity (Wald χ^2^)	Corrected Akaike information criteria (AIC)
ALBI	66.396	3068.159
APRI	25.093	3109.462
CTP	37.245	3097.310
CDS	39.791	3094.764
FIB-4	34.862	3099.693
GUCI	20.585	3113.970
Lok index	39.098	3095.457
MELD	39.644	3094.911
PALBI	38.437	3096.117
King’s score	32.170	3102.385

**Table 4:** Univariate and multivariate survival analysis in patients with hepatocellular carcinoma undergoing RFA cohort.

Overall survival	Number	Univariate analysis			Multivariate analysis		
		HR	CI	*p*	HR	CI	*p*
Age (<65/≥65 years)	227/272	1.324	1.043–1.681	0.021			
Sex (male/female)	326/173	0.836	0.647–1.055	0.126			
HBsAg (negative/positive)	262/237	0.767	0.606–0.970	0.027			
Anti-HCV (negative/positive)	296/203	1.083	0.855–1.372	0.510			
Alcoholism (no/yes)	418/81	1.126	0.816–1.554	0.471			
^a^DM (no/yes)	353/143	1.188	0.922–1.530	0.183			
Ascites (absent/present)	430/69	1.966	1.442–2.679	<0.001			
Creatinine (<1/≥ 1 mg/dL)	266/233	1.347	1.066–1.703	0.013			
ALT (≦40/>40 IU/L)	203/296	1.145	0.899–1.460	0.273			
AST (≦40/>40 IU/L)	199/300	1.643	1.281–2.108	<0.001			
^a^Alk-P (<100/≥ 100 IU/L)	260/211	1.606	1.261–2.046	<0.001			
INR of PT (<1/≥1)	127/372	1.753	1.315–2.336	<0.001			
Platelet (≥150,000/<150,000/μL)	147/352	1.763	1.325–2.346	<0.001			
Alpha-fetoprotein (<20/≥ 20 ng/mL)	259/240	1.4631	1.156–1.850	0.002			
Tumor nodules (single/multiple)	384/115	1.235	0.945–1.614	0.123			
Tumor size (≦2 cm/>2 cm)	206/293	1.541	1.206–1.968	0.001	1.635	1.269–2.106	<0.001
TTV (≦50 ml/>50 ml)	472/27	1.653	1.024–2.668	0.040			
Performance status (0/1–3)	370/129	2.309	1.780–2.996	<0.001	1.788	1.349–2.369	<0.001
Albumin-Bilirubin							
Grade 1	209		1			1	
Grade 2	261	2.306	1.779–2.989	<0.001	2.352	1.786–3.098	<0.001
Grade 3	29	5.685	3.580–8.941	<0.001	4.720	2.905–7.669	<0.001

^a^Missing of data of DM and alkaline phosphatase were 3 (0.6%) and 28 (5.6%) patients, respectively. Pooled results by multiple amputation were here. ALT, alanine aminotransferase; AST, aspartate aminotransferase; Alk-P, alkaline phosphatase; CI, confidence interval; DM, diabetes mellitus; HbsAg, hepatitis B surface antigen; HCV, hepatitis C virus; HR, hazard ratio; INR, international normalized ratio; PT, prothrombin time.

**Table 5:** Comparison of different liver function models according to the CTP score.

CTP score					
Score	5	6	7	>=8	*p*
ALBI (Mean ± SD)	−2.76 ± 0.32	−2.14 ± 0.33	−1.83 ± 0.34	−1.31 ± 0.36	<0.001
APRI (Mean ± SD)	1.08 ± 1.13	2.21 ± 2.17	2.36 ± 2.22	3.51 ± 4.06	<0.001
CDS (Mean ± SD)	5.53 ± 1.34	6.76 ± 1.46	7.33 ± 1.21	7.76 ± 1.73	<0.001
FIB-4 (Mean ± SD)	4.13 ± 3.34	7.87 ± 7.24	8.07 ± 5.16	10.49 ± 7.57	<0.001
GUCI (Mean ± SD)	1.15 ± 1.29	2.45 ± 2.46	2.86 ± 2.72	5.01 ± 6.77	<0.001
Lok index (Mean ± SD)	0.47 ± 0.20	0.66 ± 0.20	0.76 ± 0.18	0.83 ± 0.20	<0.001
MELD (Mean ± SD)	8.65 ± 2.90	9.40 ± 3.11	10.56 ± 2.57	14.41 ± 3.79	<0.001
PALBI (Mean ± SD)	−2.71 ± 0.25	−2.42 ± 0.30	−2.20 ± 0.28	−1.73 ± 0.27	<0.001
King’s score (Mean ± SD)	34.54 ± 39.30	72.21 ± 68.05	80.80 ± 79.89	134.58 ± 179.17	<0.001

**Figure 3:**
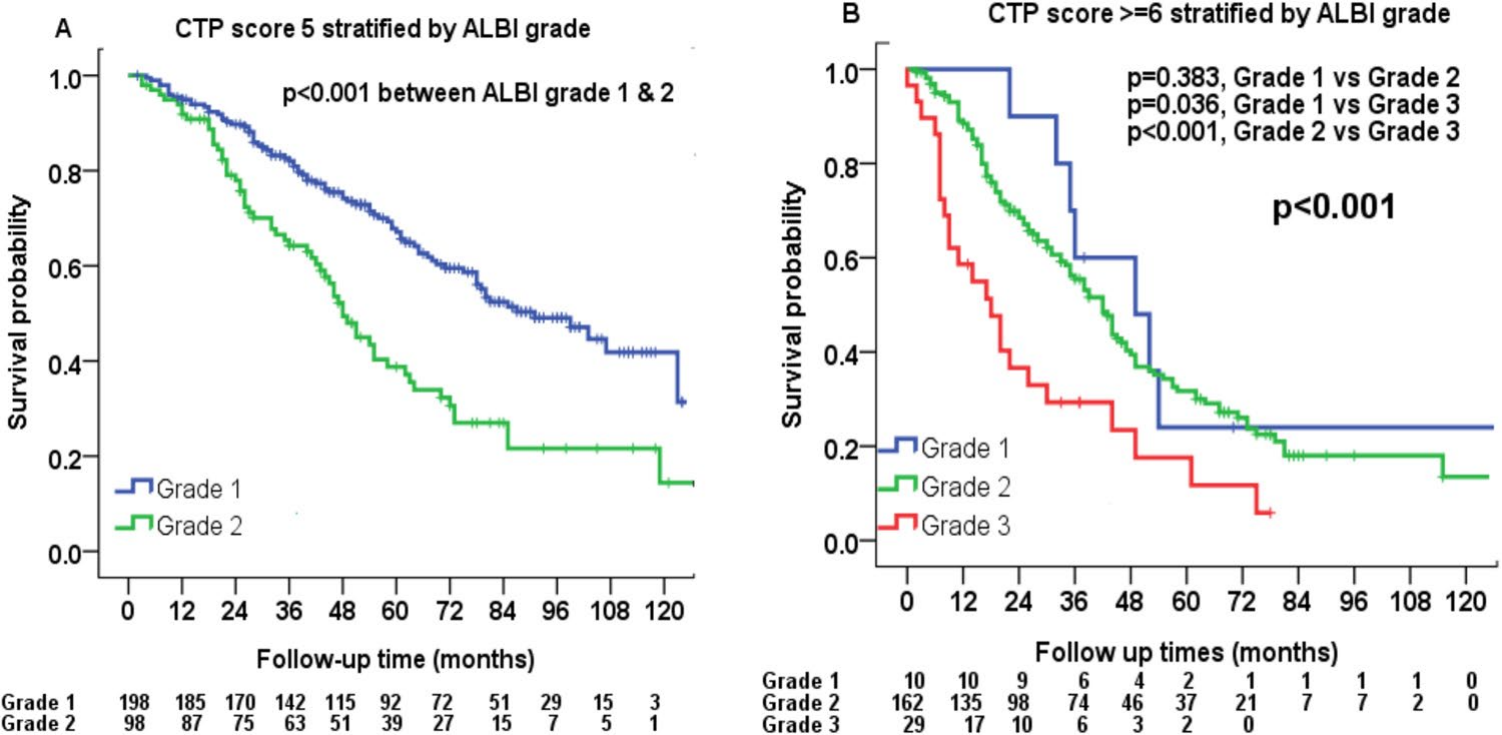
The ALBI grade is capable to classify CTP score 5 patients into two prognostic groups (ALBI grade 1 and grade 2, p < 0.001) and CTP score ≧6 into three prognostic groups (p < 0.001).

Liver function was further evaluated as stratified by the ALBI grade. For patients with CTP score 5, 198 (67%) were ALBI grade 1 and 98 (33%) were ALBI grade 2; significant survival difference was found between ALBI grade 1 vs 2 (*p* < 0.001, Fig. 3A). For patients with CTP score 6 or more, 10 (5%) were ALBI grade 1, 162 (80%) were grade 2 and 29 (15%) were grade 3; significant survival differences were identified between ALBI grade 1 vs 3 and between grade 2 vs 3 (both *p* < 0.05, Fig. 3B).

## Discussion

Liver function plays an important role in treatment and prognosis of HCC^5^. CTP classification is traditionally utilized as a surrogate model to represent liver dysfunction in HCC patients, but various new models are proposed and being used nowadays. In this study, we recruited a large, well-documented, and adequately followed-up HCC cohort. Possible prognostic predictors were examined and up to ten noninvasive liver function models were evaluated. Our results disclose that tumor status, underlying hepatic reserve and performance status are the key prognostic predictors. We also show that among the ten noninvasive models, ALBI grade is the best model to assess the severity of liver dysfunction in terms of outcome prediction for HCC patients undergoing RFA; these data confirm the ability of the predictive accuracy of ALBI grade for HCC.

The prognostic performance of the ten noninvasive models was analyzed and reveals that that ALBI, CDS and MELD are the three most accurate prognostic models to distinguish liver function. Of these models, ALBI grade has the greatest homogeneity of survival among patients within the same stage, suggesting it is a more feasible tool for prognostic prediction. In multivariate Cox analysis, patients with ALBI grade 2 had 2.3-fold and ALBI grade 3 had 4.7-fold increased risk of mortality as compared with those of ALBI grade 1. In additional to ALBI grade, we demonstrate that tumor size was closely related to the survival of HCC patients. Moreover, in accordance with previous studies^40^, the performance status, measured by Eastern Cooperative Oncology Group scale, was identified as an important predictor for survival. Taken together, the performance status, the severity of liver dysfunction and tumor burden are the hallmarks of prognostic predictors.

The CTP, MELD and ALBI are designed to assess the severity of liver dysfunction in HCC. A major disadvantage of CTP score is its arbitrarily defined, pre-determined cutoff points; the interpretation of ascites and degree of encephalopathy are also subjective^10^. Although the CTP classification has been traditionally used for decades, in this study, its AIC value was greater than the ALBI grade, suggesting the prognostic power of CTP is inferior to ALBI and some other models. Additionally, some HCC patients are not cirrhotic at the time of diagnosis^15^. The MELD score is an alternative tool used to measure liver reserve. However, serum creatinine is one of the components of MELD and this score may be less reliable in HCC patients because cancer-related cachexia might not be adequately reflected. Compared to these commonly used models, ALBI is a less subjective and widely validated prognostic model for HCC^15–17^.

Although APRI, FIB-4 index and King’s score were used to assess the severity of liver fibrosis, previously studies demonstrated that these three models have been evaluated as prognostic models for HCC^24,30,41^. Of particular interest, APRI and FIB-4 may predict recurrence of HCC. These two models were likely to associate with hepatocarcinogenesis which may account for the relation between liver fibrosis and recurrence^23,24,42,43^. Notably, other liver function models (CDS, GUCI and Lok index) have never been validated to assess the severity of liver dysfunction in HCC patients. Alternatively, the more recently proposed PALBI grade appears as an “updated” version of the ALBI; however, its usefulness cannot be confirmed in HCC patients undergoing RFA and requires more studies to establish.

The performance of ALBI grade within the same CTP score was investigated in this study. A clear advantage of the ALBI grade over CTP classification is that ALBI is equally applicable to HCC patients with minimal or no cirrhosis. In patients with CTP score 5, there were two distinct prognostic groups. This result implies that not all CTP class A patients are the same. In addition, for those with CTP scores >5, three different prognostic groups can be demonstrated by the ALBI grades. These results further display the feasibility of the ALBI grade in prognostic prediction for HCC.

The ALBI grade, based solely on serum albumin and total bilirubin, shows a better prognostic performance among the 10 models in this study. Therefore, the ALBI grade may be considered to integrate into the current HCC staging systems to further refine their prognostic power. A potential weakness of the ALBI score is that it could be influenced by albumin replacement therapy or the presence of obstructive jaundice when in some cases HCC may present with obstructive jaundice, and thus might not accurately reflect true liver functional reserve at all times.

This study has some limitations. First, the results are based on HCC patients undergoing RFA. The generalization of this study may be limited in patients receiving other therapeutic options. Second, this single-center study was performed in an area where HBV infection is prevalent; therefore, external or cross validation may be required to justify the reproducibility from other study groups. Third, as a tertiary medical center, the referral bias cannot be completely avoided.

In conclusion, our results show that the ALBI grade is the best prognostic tool among the 10 currently used noninvasive liver function models. The ALBI grade is a simple, more objective, and evidenced-based discriminatory model to represent liver reserve. Also, it is more clinically feasible because of improved prognostic power, especially in patients with minimal liver dysfunction. Incorporation of the ALBI model in different HCC staging systems needs further studies to clarify.
